# Letter to the Editor: Comparison of suprapatellar versus infrapatellar approaches of intramedullary nailing for distal tibia fractures

**DOI:** 10.1186/s13018-021-02241-8

**Published:** 2021-01-28

**Authors:** Ke Lu, Rong-xun Qian, Yi Yin

**Affiliations:** Department of Orthopaedics, Affiliated Kunshan Hospital of Jiangsu University, No. 91 West of Qianjin Road, Suzhou, 215300 Jiangsu China

Dear Editor,

We read with interest the study by Lu et al [1]. which compared clinical and functional outcomes associated with intramedullary nailing (IMN)-mediated distal tibia fracture treatment via a suprapatellar (SP) or infrapatellar (IP) surgical approach. While we have great appreciation for the work conducted by the authors in the completion of this study, there is one issue that we feel should be addressed in an effort to strengthen this article. In the “Methods” section, the authors described the ideal entry point for tibial nailing of the SP approach as being “in the intersection of tibial midline and tibial plateau articular surface,” with this point being shown in Figure 2 of their article. However, other authoritative studies have previously stated that the ideal entry point is instead located “medial to the lateral tibial spine on the anteroposterior radiograph and immediately adjacent and anterior to the articular surface as visualized on the lateral radiograph” [2, 3]. In the tibial medullary canal, the guide wire must be directed towards the central position in both planes [4]. Clearly, the description provided by Lu et al. was inaccurate, and we find that the lateral radiograph in Figure 2b shows a lower entry point. When this point is too low and the nail angle is too large, this can pose a danger to the posterior surface of the proximal tibia, especially when utilizing thick and inflexible nails [5]. The *Journal of Orthopaedic Surgery and Research* is a very influential journal, and as such, we feel obligated to point out this issue in order to ensure that readers are aware of the importance of entering the medullary canal at the appropriate point when conducting an SP approach and to ensure that the images accompanying the article represent a truly safe technique.

## Data Availability

Not applicable.

## Abbreviations

IMN: Intramedullary nailing
SP: Suprapatellar
IP: Infrapatellar
